# Microglia in radiation‐induced brain injury: Cellular and molecular mechanisms and therapeutic potential

**DOI:** 10.1111/cns.14794

**Published:** 2024-06-12

**Authors:** Yafeng Wang, Jiayu Tian, Dandan Liu, Tao Li, Yanna Mao, Changlian Zhu

**Affiliations:** ^1^ Henan Neurodevelopment Engineering Research Center for Children, Children's Hospital Affiliated to Zhengzhou University, Department of Pediatrics Henan Children's Hospital Zhengzhou Children's Hospital Zhengzhou China; ^2^ Department of Hematology and Oncology, Children's Hospital Affiliated to Zhengzhou University Henan Children's Hospital Zhengzhou Children's Hospital Zhengzhou China; ^3^ Department of Electrocardiogram, Children's Hospital Affiliated to Zhengzhou University Henan Children's Hospital Zhengzhou Children's Hospital Zhengzhou China; ^4^ Henan Key Laboratory of Child Brain Injury and Henan Pediatric Clinical Research Center, Department of Pediatrics Institute of Neuroscience and Third Affiliated Hospital of Zhengzhou University Kangfuqian Street 7 Zhengzhou 450052 None Selected China; ^5^ Center for Brain Repair and Rehabilitation, Department of Clinical Neuroscience Institute of Neuroscience and Physiology, Sahlgrenska Academy, University of Gothenburg Medicinaregtan 11 Göteborg 40530 Sweden

**Keywords:** astrocyte, cell death, microglia, neuroinflammation, neuron, radiation‐induced brain injury

## Abstract

**Background:**

Radiation‐induced brain injury is a neurological condition resulting from radiotherapy for malignant tumors, with its underlying pathogenesis still not fully understood. Current hypotheses suggest that immune cells, particularly the excessive activation of microglia in the central nervous system and the migration of peripheral immune cells into the brain, play a critical role in initiating and progressing the injury. This review aimed to summarize the latest advances in the cellular and molecular mechanisms and the therapeutic potential of microglia in radiation‐induced brain injury.

**Methods:**

This article critically examines recent developments in understanding the role of microglia activation in radiation‐induced brain injury. It elucidates associated mechanisms and explores novel research pathways and therapeutic options for managing this condition.

**Results:**

Post‐irradiation, activated microglia release numerous inflammatory factors, exacerbating neuroinflammation and facilitating the onset and progression of radiation‐induced damage. Therefore, controlling microglial activation and suppressing the secretion of related inflammatory factors is crucial for preventing radiation‐induced brain injury. While microglial activation is a primary factor in neuroinflammation, the precise mechanisms by which radiation prompts this activation remain elusive. Multiple signaling pathways likely contribute to microglial activation and the progression of radiation‐induced brain injury.

**Conclusions:**

The intricate microenvironment and molecular mechanisms associated with radiation‐induced brain injury underscore the crucial roles of immune cells in its onset and progression. By investigating the interplay among microglia, neurons, astrocytes, and peripheral immune cells, potential strategies emerge to mitigate microglial activation, reduce the release of inflammatory agents, and impede the entry of peripheral immune cells into the brain.

## Graphical abstract

Radiation induces endothelial cell damage, disrupting the blood–brain barrier, and triggering the activation of microglia and astrocytes. This activation initiates neuroinflammation and the release of inflammatory molecules, ultimately resulting in neuronal axonal damage and demyelination.

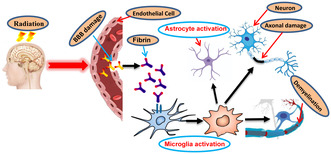

## 1 INTRODUCTION

Radiotherapy is one of the main treatments for cranial malignancies.^1^, ^2^ Advancements in technologies such as stereotactic radiotherapy and three‐dimensional conformal intensity‐modulated radiotherapy have significantly improved the prognosis for patients with cranial tumors.^3^ However, complications arising from radiotherapy still persist.^4^, ^5^ Radiation‐induced brain injury emerges as a significant and severe complication following radiotherapy for individuals with primary, secondary, or intracranial tumors.^6^, ^7^ The condition can lead to brain tissue necrosis, edema, demyelination, and subsequent cognitive and memory impairment.^8^ The precise causes of radiation‐induced brain injury remain elusive, leading to a paucity of effective and specialized treatment options. Consequently, targeting microglia emerges as a promising strategy to alleviate these consequences. Microglias, innate immune cells in the brain, have demonstrated their critical role in the development and progression of central nervous system (CNS) damage and diseases.^9^ This article comprehensively reviews recent advancements pertaining to microglial activation in radiation‐induced brain injury and explores the associated mechanisms.

## 2 RADIATION‐INDUCED BRAIN INJURY

Radiation‐induced brain injury is typically classified into three categories based on the time of occurrence following radiation exposure: acute brain injury, early delayed brain injury and late delayed injury.^10^ Acute brain injury typically arises a few days after radiotherapy, presenting mild symptoms such as headaches, feelings of nausea, and vomiting.^11^ Early delayed brain injury may surface from several weeks up to 6 months after radiotherapy, leading to potential mood changes like drowsiness and irritability.^12^ Both acute and early delayed brain injuries are generally reversible, with symptoms markedly alleviated through dehydration treatment and cranial pressure reduction.^13^ Late delayed brain injury, characterized by radiation‐induced brain necrosis, represents the most frequent clinical manifestation following radiation exposure.^14^ It manifests as either focal necrosis or diffuse white matter encephalopathy, with cerebral atrophy developing months to years following radiotherapy. MRI scans may reveal visible T1‐enhanced high signals.^8^ Late delayed brain injury is progressively irreversible and significantly impacts patients' quality of life.^12^

## 3 MICROGLIAL ACTIVATION AFTER RADIATION

### 3.1 Changes in microglial phenotypic following radiation

Microglia, essential immune cells within the CNS, exhibit distinct morphological variations categorized as ramified, hyper‐ramified, or unramified (bushy or amoeboid).^15^, ^16^ Resting microglia are highly branched and contribute to immune surveillance in the brain. However, exposure to cranial radiation induces changes in microglial cell bodies, protrusions, shape, and phagocytic activity, ultimately resulting in their activation.^17^ Study by Han et al. found that in young mice, the majority of microglia exhibited a quiescent, branched morphology in the physiological state. Following a single 8 Gy whole‐brain irradiation, 90% of microglia displayed activation and morphological alterations. Protrusions on the cell surface shortened, disappeared, and assumed a dendritic or amoeboid shape.^18^ In other studies, a notable increase in the number of amoeboid microglia in the cortex and cerebellum of juvenile mice 6 h after receiving a single whole‐brain dose of 6 Gy, gradually decreasing thereafter. No significant difference in the number of activated microglia was noted between the non‐irradiated and irradiated groups 5 days after irradiation.^15^, ^19^ These findings indicate that dynamic changes in microglial morphology and phenotype over time. Consequently, targeted intervening in microglia activation at specific time points may offer a therapeutic approach to mitigate radiation‐induced brain injury.

### 3.2 Microglia contribution to neuroinflammation

Activation of microglia triggers the release of significant amounts of inflammatory cytokines and chemokines, thereby amplifying the neuroinflammatory response.^20^, ^21^ The activation status of microglia is closely linked to the extent of neuroinflammation in the brain. At 6 h post‐radiation, the expression of anti‐inflammatory cytokines CD86, CD32, IL‐10, and CD206 decreased in activated microglia. In contrast, pro‐inflammatory cytokines, monocyte chemoattractant protein‐1 (MCP‐1), tumor necrosis factor alpha (TNF‐α), interleukin‐1beta (IL‐1β), IL‐18, and IL‐1а significantly escalated. Additionally, expression levels of IL‐6, IL‐18, MCP‐1, vascular endothelial growth factor (VEGF), and granulocyte‐macrophage colony‐stimulating factor (GM‐CSF) blatantly increased at 24 h after irradiation.^18^, ^22^, ^23^

In a model using adult male rhesus monkey subjected to fractionate whole brain irradiation, radiation‐induced brain injury was attributed primarily to cerebral vascular remodeling and neuroinflammation. Fibronectin was identified as potentially playing a role in vascular remodeling and microglia activation.^24^ Moreover, elevated expression of MCP‐1 and CD68 correlated with the clustering of activated microglia. Heightened MCP‐1 level may directly trigger microglia proliferation and activation, promoting neuroinflammation by attracting circulating monocytes into the CNS.^22^, ^25^

Hwang and colleagues demonstrated that in vitro co‐culturing of microglia with astrocytes following 15 Gy irradiation resulted in the secretion of prostaglandin E2 (PGE2), IL‐6, IL‐1β, and other pro‐inflammatory cytokines by microglia. This, in turn, mediated the phenotypic changes and astrocyte reactivity proliferation. Proliferating astrocytes were found to secrete significant amounts of VEGF.^26^ Moreover, following radiation therapy, brain tissue may become hypoxic, leading to the expression of hypoxia‐inducible factor‐1α (HIF‐1α). This stimulus prompts astrocytes to produce VEGF, which in turn increases the permeability of the blood–brain barrier (BBB). Consequently, peripheral immune cells can more easily migrate into the brain, exacerbating neuroinflammation and brain edema.^26^

Severe damage to brain endothelial cells in the late delayed stage results in platelet adhesion, smooth muscle cell proliferation, migration, arterial wall thickening, lumen narrowing or occlusion, thrombosis, ischemia, and necrosis.^27^, ^28^ This exacerbates the progression of delayed brain injury.^11^, ^29^ In addition, exposure to 10 Gy of whole‐brain radiation in mice resulted in only a portion of microglia exhibiting activation, resulting in a varied response after 1 week.^22^ However, predominantly activated microglia with enlarged cell bodies were observed 2 months following irradiation, engaging in the engulfment of dead cells and damaged neurons.^11^

### 3.3 Interactions among microglia, neurons, astrocytes, and pericytes after radiation

**FIGURE 1:**
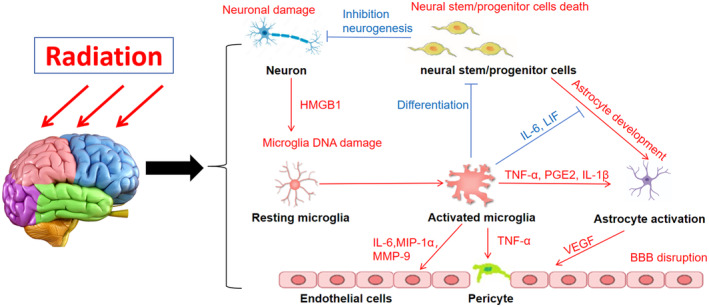
Overview the intricate interactions among microglia, neurons, astrocytes, and pericytes following cerebral radiation. Neurons secrete alarmin molecules (e.g., HMGB1) in response to the cellular damage caused by radiation, initiating microglial activation. Simultaneously, radiation‐induced DNA damage within microglia contributes to their activation. The activated microglia release proinflammatory molecules, hindering neural stem cell differentiation and adversely affecting neurogenesis and astrocyte development. In the presence of activated microglia, inflammatory mediators like TNF‐α, IL‐1, and prostaglandin E2 (PGE2), can activate astrocytes. Activated astrocytes, in turn, produce significant quantities of VEGF, enhancing the permeability of the blood–brain barrier (BBB). Additionally, inflammatory agents such as TNFα, IL‐6, MIP‐1α, and MMP‐9 induce pericyte migration and redistribution, upregulating endothelial adhesion expression. This cascade of events leads to BBB permeabilization and infiltration of peripheral immune cells into the brain parenchyma.

Microglia, neurons, astrocytes and pericytes exhibit the capacity to release inflammatory cytokines and chemokines in response to radiation‐induced brain injury (Figure 1).^30^, ^31^ These cellular interactions contribute to the amplification of neuroinflammation.^23^ During the neuroinflammatory process, neurons communicate with microglia by secreting high mobility group box‐1 (HMGB1), which binds to Toll‐like receptor 4 (TLR4) on the membrane, indirectly activating microglia.^32^ Conversely, activated microglia recognize calreticulin on neuronal membranes surface using low‐density lipoprotein receptor‐related protein, leading to the neuronal phagocytosis by microglia and consequent impairment of cognitive function.^33^

Under physiological conditions, neural stem/progenitor cells differentiate into neurons. However, microglia release IL‐6 and leukemia inhibitory factor (LIF), activating the JAK/STAT, and mitogen‐activated protein kinase (MAPK) pathways and promoting the differentiation of neural stem/progenitor cells into astrocytes. This process results in reactive gliosis proliferation and the formation of glial scars.^34^ Furthermore, the release of pro‐inflammatory cytokines such as PGE2 and IL‐1β by microglia following radiation exposure is identified as a key contributor to phenotypic alterations and reactive gliosis proliferation in astrocytes.^26^ Studies have shown that activated microglia release various inflammatory mediators, including IL‐1α, TNF‐α, and complement 1q, which can induce neurotoxic A1‐type astrocytes.^35^, ^36^ Additionally, reactive astrocytes can secrete IL‐6, TNF‐α, interferon‐gamma (IFN‐γ), and complement factor C3, impacting the phenotype, motility, and phagocytic functions of microglia.^37^ Furthermore, radiation activates the MAPK, activator protein 1 (AP‐1), and NF‐κB signaling pathways, resulting in increased expression of inflammatory cytokines IL‐1β, IL‐6, and the inflammatory mediator cyclooxygenase‐2 (COX‐2) in astrocytes, thereby further exacerbating their inflammatory phenotype.^38^ Consequently, the interplay between microglia and astrocytes post‐irradiation synergistically promotes neuroinflammation.

Astrocytic end‐feet and pericytes are anatomically connected, preserving tight junctions of endothelial cells and capillary stability under physiological conditions.^39^ In cases of radiation‐induced brain injury, astrocytes produce significant amount of VEGF, elevating the permeability of the BBB and exacerbating neuroinflammation.^40^ Treatment of patients with radiation‐induced brain injury using a VEGF antibody resulted in a 93% decrease in brain tissue necrosis and improvement in brain tissue edema in 64% of cases, suggesting antagonistic VEGF as a promising treatment option.^41^ Another study observed a decrease in perivascular cells in lesions of patients with radiation‐induced brain injury, hypothesizing that this may contribute to BBB impairment.^42^ The hypothesis that astrocytes induce fibronectin synthesis in pericytes, playing a role in basement membrane formation during inflammatory angiogenesis, requires further investigation.^43^

Under physiological conditions, pericytes and endothelial cells secrete CX3CL1, effectively inhibiting microglia activation and maintaining vascular stability. However, exposure to stress factors such as radiation, inducing neuroinflammation, leads to secretion of different cytokines by microglia and pericytes, resulting in their interaction. Activated microglia release TNFα, promote pericyte migration and redistribution, and secrete IL‐6, macrophage inflammatory protein‐1α (MIP‐1α), and matrix metalloproteinase‐9 (MMP‐9).^44^ Conversely, both IL‐6 and MIP‐1α have the potential to stimulate microglial activation and aggregation, whereas MMP‐9 impairs vascular integrity and exacerbates the progression of neuroinflammation.^45^ Endothelial cells exhibit relatively resistant to radiation,^46^ attributed to fractalkine (FKN) secretion via NF‐κB pathway activation. FKN binds to CX3CR1 on microglia, prompting their polarization towards the M2 phenotype, thus mitigating radiation‐induced brain injury.^47^ Radiation induces increased reactive oxygen species (ROS) production in cerebral endothelial cells.^48^ Additionally, endothelial cells express radiation‐induced senescence‐associated secretory phenotype (SASP),^27^ marked by heightened production of pro‐inflammatory molecules, cytokines, chemokines, and MMPs, fostering neuroinflammation and microglial activation.^49^ The intricate interplay among vascular endothelial cells, pericytes, and microglia underscores the importance of maintaining vascular microenvironment homeostasis and balance in ameliorating radiation‐induced brain injury.

## 4 SIGNAL PATHWAYS OF MICROGLIA IN RADIATION‐INDUCED BRAIN INJURY

The pathogenesis of radiation‐induced brain injury is subject to various hypotheses, including vascular damage, neuroinflammation, and demyelination.^10^, ^50^, ^51^ Of these, neuroinflammation emerges as a significant contributor to radiation‐induced brain injury. Radiation inflicts damage upon CNS cells, potentially inciting neuroinflammation, which in turn can exacerbate brain injury, possibly triggering a cascade of acute and chronic inflammatory reactions in the brain mediated by glial cells.^12^, ^52^ Microglial activation stands out as a primary factor in brain neuroinflammation.^53^ yet the precise mechanism by which radiation prompts microglial activation in radiation‐induced brain injury remains elusive.^53^ Multiple signaling pathways likely contribute to microglial activation and the progression of radiation‐induced brain injury.

### 4.1 Toll‐like receptors (TLRs) signaling pathway

The signaling pathway pf TLRs involves recruitment of specific adaptor molecules, which activates the transcription factors NF‐κB. This factor regulates the expression of various inflammatory cytokine genes.^54^ TLRs are type I transmembrane glycoproteins that play a crucial role in the immune response by specifically recognizing pathogen‐associated molecular patterns (PAMPs) and damage‐associated molecular patterns (DAMPs).^55^, ^56^, ^57^ DAMPs refer to changes occuring at the damage site immediately following the onset of a various CNS disorders, including radiation‐induced brain injury.^8^, ^58^, ^59^ Released DAMPs are detected by pattern recognition receptors, initiating immune responses.^60^ Numerous DAMPs are expressed within the CNS of healthy individuals and are subsequently released after injury, triggering an inflammatory reaction.^55^ Typical DAMPs include adenosine triphosphate (ATP), HMGB1, and IL‐33.^61^, ^62^ ATP and IL‐33 trigger microglia to release chemokine ligand 3 (CCL3), while astrocytes produce chemokine ligand 2 (CCL2), attracting neutrophils and monocytes to the injury site.^63^, ^64^ HMGB1 strongly stimulates astrocytes to secrete various chemokines, including neutrophil chemokines (CXCL1, CXCL2, and CCL3) and T‐cell chemokines (CXCL1, CCL2, CCL5, and CCL20).^65^ Meanwhile, HMGB1 binds to TLR4, enhancing the migration, proliferation and differentiation of immune cells.^66^ Consequently, radiation‐induced brain injury triggers the release of DAMPs, promoting the migration of neutrophils, monocytes, and other inherent immune cells, provoking an innate immune response and directly or indirectly triggering an adaptive immune response.

### 4.2 MAPK‐related signaling pathways

P38 MAPK, a member of the MAPK family, plays a crucial role in cellular stress responses.^67^ Its activation is considered to be a crucial factor in radiation‐induced brain injury, triggering inflammatory responses that lead to apoptosis and damage in brain tissue after radiation.^68^ In the mouse brain, the activation of the p38 MAPK pathway is closely linked to white matter damage.^69^ Selective neural *Atg7* knockout reduced the severity of radiation‐induced brain injury by inhibiting the activation of the p38 MAPK pathway.^19^ This pathway is closely associated with neuroinflammation and apoptosis in radiation‐induced brain injury.^12^ The p38 MAPK pathway is known for its regulatory function in neuroinflammation, thereby activating the production of inflammatory mediators.^70^ This process contributes to neuroinflammation, disrupting normal CNS function. Apoptosis, a critical feature of radiation‐induced brain injury is promoted by the p38 MAPK signaling pathway, activating apoptosis‐related proteins, including caspase family proteins.^71^ This mechanism potentially contributes to neural damage and cell death. Xu et al. suggests the involvement of the p38 MAPK pathway in the impact of exogenous ATP, via the P2X7 receptor, on radiation‐induced brain injury. Exogenous ATP exacerbates radiation‐induced brain damage by activating microglia and intercellular signaling of the p38 MAPK pathway.^63^ Hwang and colleagues further highlight the significance of the p38 MAPK pathway in radiation‐induced brain injury, revealing its activation's role in astrogliosis via microglia activation.^26^ These findings underscore the importance of the p38 MAPK pathway in radiation‐induced brain injury, revealing a close association with neuroinflammation, apoptosis, and intercellular signaling.

MAPK/ERK kinase (MEK) signaling pathway plays a vital role in regulating numerous cellular activities.^72^ Through phosphorylation, it activates extracellular signal‐regulated kinase (ERK) to transmit signals from external stimuli, exerting crucial control over cellular processes,^73^ Deng et al demonstrated that that radiation exposure induces c‐JUN activation in microglial cells, dependent on the MEK1‐ERK1/2 signaling pathway.^74^ This MEK/ERK/c‐JUN signaling pathway is significant in the biological effects of radiation and plays a critical role in neuroprotection. Research on sodium felluic acid's neuroprotective effects on the rat hippocampus revealed that activating this pathway alleviates neuropathic damage and enhances neurological recovery.^75^ Conversely, Bose et al investigated the impact of CCL2 on BV2 microglia migration, exploring potential signaling pathways.^76^ The MEK/ERK/c‐JUN signaling pathway may significantly contribute to microglial activation and inflammation. Additionally, this signaling pathway is relevant to regulating cell survival and apoptosis. Suzuki et al. found that P2X7 receptor‐activated microglia release tumor necrosis factor with neuroprotective properties, suggesting a potential role in the neuroprotective via the MEK/ERK/c‐JUN signaling pathway and other pathways.^77^

### 4.3 Notch signaling pathway

The Notch signaling pathway is crucial for diverse biological processes, facilitating cell–cell interaction and regulating cell proliferation, differentiation, and inflammatory responses.^78^, ^79^, ^80^ In radiation‐induced brain injury, Notch signaling pathway plays a significant role in neuroinflammation, neuronal damage, and vascular remodeling. Microglia play a pivotal role in this process, with their activation being influenced by Notch signaling.^81^, ^82^ Notch‐1 signaling, via the NF‐κB signaling pathway, is implicated in microglial activation, underscoring the pathway's importance emphasizing in inflammation regulation.^83^ Mutations in the Notch signaling pathway impact response to radiation therapy, highlighting its significance in brain injury regulation.^84^ Additionally, Notch signaling pathway is integral in the proliferation and differentiation of neural stem cells,^85^ with potential applications in neural repair following radiation‐induced brain injury.^86^ Furthermore, Notch signaling pathway is associated with cerebrovascular changes, controlling the excessive growth of smooth muscle cells induced by radiation, potentially causing pathological adjustments in cerebral vasculature.^82^ In brief, the Notch signaling pathway plays diverse roles in radiation‐induced brain injury, regulating microglia activation, influencing the response, governing neural stem cell proliferation and differentiation, participating in cerebral vascular reconstruction, and regulating neuroinflammation.

### 4.4 NF‐κB signaling pathway

The activation of NF‐κB signaling pathway plays crucial roles in various biological processes, including immune response, inflammation, and apoptosis.^87^, ^88^ Radiation exposure triggers inflammatory response in radiation‐induced brain injury, releasing cytokines, such as TNF‐α, IL‐1β, and IL‐6.^15^, ^19^ Following irradiation, the NF‐κB p65 subunit underwent nuclear translocation, and there was a significant upregulation of essential modulator (NEMO) and a significant decrease in inhibitor of NF‐κB regulation‐α.^89^ This suggests NF‐κB activation triggers microglia activation and inflammatory factors release.^90^, ^91^ Corilagin impedes NF‐κB pathway activation by STAT3‐related pathway, reducing inflammatory cytokine expression.^92^, ^93^ Furthermore, NF‐κB signaling pathway activation is implicated in obstructing apoptosis by potentially regulating the expression Bcl‐2 expression. Suppressing the PIDD‐C/NF‐κB transcription pathway can hinder microglia activation, involving expression of PIDD, a keyregulator between the NF‐κB transcription pathway and radiation‐induced apoptosis.^94^ NF‐κB activation is linked to immune cell activation and neuroprotection. Regulating NF‐κB signaling can reduce excessive activation of immune cells, protecting neural tissue. PPARδ transinhibits NF‐κB through physical interaction with the p65 subunit, preventing PKCα/MEK1/2/ERK1/2/AP‐1 pathway activation by inhibiting radiation‐induced intracellular reactive oxygen species generation.^95^ Kukoamine A reduces nuclear translocation of the NF‐κB p65 subunit and c‐jun phosphorylation induced by whole‐brain irradiation while enhancing PPARδ expression.^96^

### 4.5 ATP/P2X7R signaling pathway

The ATP/P2X7R signaling pathway is crucial role in cell death, inflammation, and immune response, and holding significance in both radiation therapy and neurological disorders.^97^, ^98^ Research on the P2X7 receptor suggests its pivotal function in predicting glioma sensitivity towards radiation therapy and patient median survival,^99^ highlighting its intimate correlation with the glioma response to radiation therapy. Activation of the P2X7 receptor amplifies cell death in radiosensitive human glioma cell lines.^100^ In radiation‐induced brain injury, Xu et al suggest that P2X7 receptors activate microglia, mediating extracellular signaling that contributes to brain injury.^63^ Another study underscores the importance of P2X7 receptors in cognitive dysfunction resulting from HIV protein gp120,^101^ indicating their potential role in nervous system inflammation and neurodegenerative diseases. In addition, the loss of P2X7 receptor observed in radiotherapy‐induced saliva production inhibition underscores their significant involvement in the negative effects of radiotherapy.^102^ Astaxanthin (AST) research on epileptic rats demonstrates its ability to regulate ATP/P2X7R signaling, reducing neuroinflammation.^86^ This suggests the crucial function of the ATP/P2X7R signaling pathway in various disease contexts, including neurodegenerative diseases, radiation therapy‐induced brain damage, and other inflammatory conditions.

## 5 SUMMARY

The intricate microenvironment and molecular mechanisms linked to radiation‐induced brain injury involve crucial roles immune cells in both its onset and progression. By investigating the interplay among microglia, neurons, astrocytes, and peripheral immune cells, potential strategies emerge to mitigate microglial activation, diminish inflammatory agents release, and impede the entry of peripheral immune cells into the brain. These interventions have the potential to alleviate cognitive dysfunction and offer novel therapeutic options for managing radiation‐induced brain injury. Additionally, regulating microglial polarization towards the M2 type holds promise in minimizing neuroinflammation and promoting the reparation of neural tissues, presenting a novel avenue for research treating radiation‐induced brain injury. Directing therapeutic interventions towards microglia could present an innovative strategy, substantially enhancing the long‐term quality of life for survivors of cranial tumor radiotherapy.

## AUTHOR CONTRIBUTIONS

CZ devised the review. YW and JT reviewed the literatures and wrote the manuscript drafts. YW, DL, TL, YM, and CZ carried out writing‐review and editing. YM and CZ substantially contributed to the literature review and the writing of this manuscript. All authors have read and agreed to the published version of the manuscript.

## FUNDING INFORMATION

This work was supported by the National Natural Science Foundation of China (U21A20347, 82003396), the Henan Medical Science and Technique Foundation (SBGJ202303048, SBGJ202301009), the Henan Provincial Science and Technology Research Project (222102310431, GZS2023003), Swedish Cancer foundation (201121Pj, 233115Pj), Childhood Cancer foundation (PR2021‐0020), Adlerbert Research Foundation (2023‐684) and Jacobsons Donationsfond (2023‐141).

## CONFLICT OF INTEREST STATEMENT

The authors declares that there is no conflict of interest regarding the publication of this paper.

## Data Availability

All the data supporting the findings of this study are included in this article.
